# Whole-genome association studies on alcoholism comparing different phenotypes using single-nucleotide polymorphisms and microsatellites

**DOI:** 10.1186/1471-2156-6-S1-S130

**Published:** 2005-12-30

**Authors:** Liang Chen, Nianjun Liu, Shuang Wang, Cheongeun Oh, Nicholas J Carriero, Hongyu Zhao

**Affiliations:** 1 Department of Epidemiology and Public Health, Yale University, New Haven, CT 06520, USA; 2 Division of Biostatistics, Department of Preventive Medicine, University of Medicine and Dentisry of New Jersey, Newark, NJ 07101, USA; 3 Department of Biostatistics, University of Alabama at Birmingham, Birmingham, AL 35294, USA; 4 Department of Biostatistics, Mailman School of Public Health, Columbia University, New York, NY 10032, USA; 5 Department of Computer Science, Yale University, New Haven, CT 06520, USA; 6 Department of Genetics, Yale University, New Haven, CT 06520, USA

## Abstract

Alcoholism is a complex disease. As with other common diseases, genetic variants underlying alcoholism have been illusive, possibly due to the small effect from each individual susceptible variant, gene × environment and gene × gene interactions and complications in phenotype definition. We conducted association tests, the family-based association tests (FBAT) and the backward haplotype transmission association (BHTA), on the Collaborative Study of the Genetics of Alcoholism (COGA) data provided by Genetic Analysis Workshop (GAW) 14. Efron's local false discovery rate method was applied to control the proportion of false discoveries. For FBAT, we compared the results based on different types of genetic markers (single-nucleotide polymorphisms (SNPs) versus microsatellites) and different phenotype definitions (clinical diagnoses versus electrophysiological phenotypes). Significant association results were found only between SNPs and clinical diagnoses. In contrast, significant results were found only between microsatellites and electrophysiological phenotypes. In addition, we obtained the association results for SNPs and microsatellites using COGA diagnosis as phenotype based on BHTA. In this case, the results for SNPs and microsatellites are more consistent. Compared to FBAT, more significant markers are detected with BHTA.

## Background

Alcoholism is a serious public health problem. Genetic variants underlying alcoholism have been difficult to identify for many reasons, including issues with diagnoses, disease heterogeneity, gene × gene and gene × environment interactions. These reasons present a great challenge for human geneticists to identify genes associated with alcoholism susceptibility.

Recently, great efforts have been devoted to conducting genome-wide analysis on a large number of families to map genes for alcoholism. For example, the Collaborative Study of the Genetics of Alcoholism (COGA) collected 1,614 family members, including alcoholic people and their relatives. For each individual, a total of 15,840 single-nucleotide polymorphism (SNP) markers from Affymetrix and Illumina and 328 microsatellite markers have been genotyped. Both COGA diagnosis and DSM-IV diagnosis are used to define each person's phenotype. In addition, the electrophysiological phenotypes are tested by the Visual Oddball experiment with event-related potential (ERP) records and the Eyes Closed Resting electroencephalogram (EEG) experiment. The associations between alcoholism and ERP and EEG have been reported in several published papers [1,2].

In this paper we perform family-based association tests (FBAT) [3] based on SNPs and microsatellites, using both clinical diagnosis phenotypes and electrophysiological phenotypes, to identify genetic variants associated with alcoholism in the COGA dataset. In order to consider possible gene × gene interactions, we also perform backward haplotype transmission association (BHTA) tests [4] based on SNPs and microsatellites using COGA diagnosis phenotype.

## Methods

### FBATs for different phenotypes and different markers

The original transmission disequilibrium test (TDT) was proposed to test genetic linkage in the presence of association between a candidate marker and disease phenotype by comparing, among heterozygous parents, the total number of a specific allele transmitted to the affected offspring with what would be expected under the null hypothesis [5]. Laird and colleagues have extended the original TDT to a comprehensive association analysis approach called FBAT [3], which is implemented in the FBAT program [6]. Conditioning on the sufficient statistics for any nuisance parameters, the expected allele distributions are obtained under the null hypothesis of no association. This method avoids confounding due to model misspecification and admixture or population stratification. In this paper, we use FBAT to test association and linkage between genetic markers and phenotypes in the COGA dataset. The phenotypes analyzed include COGA diagnosis, DSM-IV diagnosis, and ERP electrophysiological phenotypes. The genetic markers analyzed include SNPs and microsatellites.

FBAT was performed for every SNP marker (15,406) and microsatellite marker (315) except those on the X chromosome; these markers were tested individually. All of the family members in COGA were included in the study. Individuals who never drink alcohol or have some symptoms but do not meet the diagnosis criteria were considered as having unknown disease phenotype. According to the *t*-tests between purely unaffected and affected unrelated persons, ttdt1 and ttdt4 channels in the ERP dataset have their *p*-values less than 0.1. ttdt1 corresponds to electrodes placed on the scalp location FP1, which is the far frontal left side channel, and ttdt4 corresponds to electrodes placed on the scalp location PZ, which is the parietal midline channel. These two measures are used as quantitative traits in FBAT. The offset values μ for COGA diagnosis and DSM-IV diagnosis results are set to be 0, and the offset values μ for the electrophysiological phenotypes are set to be the sample means. Here, μ is a nuisance parameter, and the misspecification of μ will not bias the test (different values of μ for COGA diagnosis and DSM-IV diagnosis (0.2 and 0.5) have been tested and similar results are obtained). The additive models are used for the genotype coding.

Efron's local false discovery rate method [7] was applied to the FBAT results to identify significant markers after multiple comparison adjustments. This method is implemented in the R package "locfdr" [8]. Let z be the test statistics or the transformed *p*-values (z = Φ^-1^(p), where Φ indicates the standard normal cumulative function). Let f(z) be the density function of z. We assume f(z) = p_0_f_0_(z)+p_1_f_1_(z), where f_0_(z) is the density function for non-significant markers and f_1_(z) is the density function for significant markers. The natural spline method is applied to estimate f(z). f_0_(z) is the theoretical null distribution (the standard normal distribution) or the empirical null distribution that is a normal distribution with mean and variance estimated from the central part of the f(z) fit. The local false discovery rate is defined by f_0_(z)/f(z), which is focusing on density. Benjamini and Hochberg's false discovery rate [9] corresponds to the "tail-area" of the local false discovery rate. The false discovery rate of z can be written as the weighted average of local false discovery rate of z_i _(z_i _is from z to ∞). Therefore, when we use a local false discovery rate 0.1 as our criterion, the corresponding false discovery rate should be less than 0.1. For SNPs, we used z as the test statistics because the distribution of the test statistic is approximately *N*(0,1) and chose f_0_(z) as the theoretical null. We used a full range of z to estimate f(z) and 5 degrees of freedom for splines and 60 breaks for the histogram counts. For microsatellites, we used the transformed *p*-values as z because the distribution of the test statistics is not approximately *N*(0,1) and choose f_0_(z) as the estimated empirical null. We used the full range of z to estimate f(z) and 5 degrees of freedom for splines and 60 breaks for the histogram counts. Markers with a local false discovery rate <0.1 were included in the summary results.

### BHTA approach for different markers

Another extension of the original TDT is the BHTA algorithm [4]. In BHTA, the inferred haplotypes are treated as alleles in TDT. The haplotypes transmitted to the affected offspring are compared with the expected haplotype distribution among all the offspring, where haplotype has a generalized definition in this procedure [4]. For BHTA, a small number of markers are randomly selected each time to construct a candidate haplotype. A backward selection algorithm is then used to screen out unimportant markers one by one until only the important markers associated with the trait remain. The sampling is repeated many times and the markers returned most often are considered as the associated markers. BHTA may take the interactions between markers into account because it considers haplotype information, and BHTA is computationally efficient for a whole-genome scan study. In this paper, we use BHTA to identify markers associated with disease phenotype for the COGA dataset accounting for both joint and marginal effects.

The imputation of missing genotypes and the inference of haplotypes given multilocus unphased genotypes were performed according to the procedure described in Lo and Zheng [10]. There are 266 trios with an affected child in the study. The families with more than one affected child were partitioned into multiple trios, and this extension is validated by Lo and Zheng [4]. Microsatellites were dichotomized according to their repeat numbers with the probability of "allele 0" as close to 0.5 as possible. Based on COGA diagnosis, for the 15,406 SNPs, we sampled 30 markers each time and repeated the sampling 200,000 times. For the 315 microsatellites, we sampled 30 markers each time and repeat the sampling 20,000 times. For each sampling, the haplotype information based on the 30 markers was considered and the unimportant markers were deleted. The returned frequency for each marker was recorded.

The local false discovery rate (fdr) method [7] was applied to the returned frequencies to separate the significant markers and the non-significant markers. We used the returned frequencies as z and chose f_0_(z) as the estimated empirical null. The full range of z was used to estimate f(z) and 5 degrees of freedom were used for splines and 60 breaks were used for the histogram counts. Local fdr = 0.1 was chosen as the selection criterion, which corresponds to a returned frequency of 310 for SNPs and 908 for microsatellites.

## Results

### FBAT results

A total of 6 SNPs were found to be associated with COGA diagnosis at local fdr = 0.1. They are located on chromosomes 3, 9, 13, 16, and 20. Four SNPs were associated with DSM-IV diagnosis at fdr = 0.1. They are located on chromosomes 1, 6, 9, and 11. SNP tsc0124879 on chromosome 9 is common for these two clinical diagnoses. For ERP, no significant SNP was detected at fdr = 0.1 for either the ttdt1 or ttdt4 channel. For microsatellites, D16S3253 on chromosome 16 was found to be associated with ttdt1 channel at fdr = 0.1. No significant microsatellites were detected at fdr = 0.1 for either COGA diagnosis or DSM-IV diagnosis. The above results are summarized in Table 1.

### BHTA results

BHTA is only applied to COGA diagnosis in this study. For SNPs, using a local fdr = 0.1 as the criterion that corresponds to a returned frequency of 310, 23 SNPs were found to be significant with respect to the COGA diagnosis. Among these 23 SNPs, 3 are on chromosome 9, 3 on chromosome 13, 2 on chromosomes 1, 5, 6, and 14, and the other SNPs are on chromosomes 3, 4, 7, 8, 10, 15, 16, 18, and 20. SNP tsc0271621 on chromosome 13 was found to be significant based on both FBAT and BHTA. These results are summarized in Table 2. For microsatellites, using a local fdr = 0.1 as the criterion that corresponds to a returned frequency of 908, GATA175H06 on chromosome 9 and D2S2370 on chromosome 2 are significant.

## Discussion

We have obtained the FBAT results for different phenotypes for SNPs and microsatellites. The results for COGA diagnosis and DSM-IV diagnosis are similar because 27 out of the top 50 markers are shared between these two diagnoses (data not shown). However, the results for clinical diagnoses are different from those for electrophysiological phenotypes. For the two clinical diagnoses, 6 and 4 significant SNPs were found at fdr = 0.1, with no significant microsatellites. Among the significant SNPs, SNP tsc0124879 on chromosome 9 is common for the two clinical diagnoses. For the ERP channel ttdt1, one significant microsatellite (D16S3253) was found at fdr = 0.1, with no significant SNPs. Because the SNP scan has a higher resolution than the microsatellite scan, it is more likely that we would identify more significant SNPs in this study due to the better coverage in terms of linkage disequilibrium. However, the underlying reasons for the different results for the clinical phenotypes and electrophysiological phenotypes are unclear. One possible reason may be that the electrophysiological phenotypes are associated with disturbed cognitive processing, which involves not only alcoholism but also other psychiatric behaviors. There are 23 significant SNPs and 2 significant microsatellites in the BHTA results. Among the 3 significant SNPs on chromosome 9, tsc0607689 (23.9832 cM) is close to tsc0607688 (23.9834 cM). Among the 3 significant SNPs on chromosome 13, tsc1102168 (11.136 cM) is close to tsc1102169 (11.1366 cM). For microsatellites, GATA175H06 on chromosome 9 (21.5 cM) is significant. It is close to significant SNPs tsc0607689 (23.9832 cM) and tsc0607688 (23.9834 cM). The number of significant SNPs (23) in the BHTA study is larger than that in the FBAT study (6 or 4). In principle, BHTA may be able to capture gene × gene interactions, including genes that do not have marginal effects but have significant interactions with other genes. Chromosome 9 is mapped to alcoholism for both SNPs and microsatellites. In addition, we have a significant marker tsc0271621 on chromosome 13 for both FBAT and BHTA.

## Conclusion

In this study, we compared the use of different phenotypes (clinical phenotypes and electrophysiological phenotypes) and different types of genetic markers (SNPs and microsatellites) to identify genetic variants underlying alcoholism in the framework of family-based association tests. Significant SNPs were found for clinical phenotypes and a significant microsatellite was found for ERP phenotypes. There is little overlap of significant regions identified based on two different types of markers. Compared to FBAT, we have detected more significant SNPs using BHTA. For BHTA, the microsatellite results are consistent with the SNP results according to their close genetic positions (within 3 cM). Both FBAT and BHTA reveal that SNP tsc0271621 is significant.

## Abbreviations

BHTA: Backward haplotype transmission association

COGA: Collaborative Study on the Genetics of Alcoholism

EEG: Electroencephalogram

ERP: Event-related potential

FBAT: Family-based association test

fdr: False discovery rate

GAW14: Genetic Analysis Workshop 14

SNP: Single-nucleotide polymorphism

TDT: Transmission disequilibrium test

## Authors' contributions

LC participated in the design of the study, performed the analysis, and drafted the manuscript. NL, SW, and CO participated in the design of the study. NJC participated in the programming. HZ supervised the study, participated in its design, and helped to draft the manuscript. All authors read and approved the final manuscript.

## Figures and Tables

**Table 1:** FBAT results for different genetic markers and different phenotypes at local false discovery rate 0.1

	Name	Chromosome	Local false discovery rate	Physical position	Genetic position
Significant SNPs for COGA diagnosis	tsc0124879	9	0.00192	94365247	103.211
	tsc1750530	16	0.00935	40509969	59.8297
	tsc0515272	3	0.0270	153432854	164.236
	tsc0060446	20	0.0670	12182481	35.4473
	tsc0271621	13	0.091	63868120	60.1748
	tsc0056748	13	0.095	76951496	73.9934

Significant SNPs for DSM-IV diagnosis	tsc0124879	9	0.0184	94365247	103.211
	tsc0569292	11	0.0385	5143142	6.78451
	tsc1177810	1	0.0542	81549852	105.535
	tsc0808295	6	0.0660	23774023	47.1522

Significant Microsatellite for ttdt1 channel	D16S3253	16	0.0486		82.7

**Table 2:** BHTA results for different markers using COGA diagnosis phenotype at local false discovery rate 0.1

	Name	Chromosome	Returned Frequency	Physical position	Genetic position
Significant SNPs	tsc0051201	5	445	123934709	129.079
	tsc0607688	9	423	11181543	23.9834
	tsc0047552	7	408	14718190	28.405
	tsc0511137	8	400	3989846	7.47656
	tsc1056525	18	399	23369689	48.1751
	tsc1458383	6	386	63408725	80.7566
	tsc0342869	4	381	191320090	204.47
	tsc0183603	5	380	2432756	4.28753
	tsc1084268	20	370	57200560	98.5039
	tsc0694296	1	364	4349628	8.0634
	tsc1212413	16	355	46150212	71.101
	tsc0271621	13	316	63868120	60.1748
	tsc0607689	9	434	11181529	23.9832
	tsc0016057	14	410	90209951	94.9861
	tsc1102168	13	401	22774216	11.136
	tsc1102169	13	399	22774326	11.1366
	tsc0050133	6	391	131397208	130.741
	tsc1443434	15	384	18511390	3.61027
	tsc0502368	9	381	112556523	125.36
	tsc1195531	14	374	18383782	5.9575
	tsc0954978	1	368	149990102	145.896
	tsc0045109	3	360	123785701	134.022
	tsc0414849	10	332	93647411	112.752

Significant Microsatellites	GATA175H06	9	1856		21.5
	D2S2370	2	1085		184.3
